# Management of MET-Driven Resistance to Osimertinib in *EGFR*-Mutant Non-Small Cell Lung Cancer

**DOI:** 10.3390/genes16070772

**Published:** 2025-06-30

**Authors:** Panagiotis Agisilaos Angelopoulos, Antonio Passaro, Ilaria Attili, Pamela Trillo Aliaga, Carla Corvaja, Gianluca Spitaleri, Elena Battaiotto, Ester Del Signore, Giuseppe Curigliano, Filippo de Marinis

**Affiliations:** 1 Department of Oncology and Haemato-Oncology, University of Milan, 20122 Milan, Italy; panagiotisagisilaos.angelopoulos@ieo.it (P.A.A.); elena.battaiotto@ieo.it (E.B.);; 2 Division of Thoracic Oncology, European Institute of Oncology, IRCCS, 20141 Milan, Italy; ilaria.attili@ieo.it (I.A.); pamela.trilloaliaga@ieo.it (P.T.A.); carla.corvaja@ieo.it (C.C.); gianluca.spitaleri@ieo.it (G.S.); ester.delsignore@ieo.it (E.D.S.); filippo.demarinis@ieo.it (F.d.M.); 3 Division of Early Drug Development, European Institute of Oncology, IRCCS, 20141 Milan, Italy

**Keywords:** osimertinib, *EGFR*, *MET*, resistance, ADC

## Abstract

Epidermal growth factor receptor (*EGFR*) mutations occur in approximately 10–20% of Caucasian and up to 50% of Asian patients with oncogene-addicted non-small cell lung cancer (NSCLC). Most frequently, alterations include exon 19 deletions and exon 21 L858R mutations, which confer sensitivity to EGFR tyrosine kinase inhibitors (TKIs). In the last decade, the third-generation EGFR-TKI osimertinib has represented the first-line standard of care for EGFR-mutant NSCLC. However, the development of acquired mechanisms of resistance significantly impacts long-term outcomes and represents a major therapeutic challenge. The mesenchymal–epithelial transition (*MET*) gene amplification and MET protein overexpression have emerged as prominent EGFR-independent (off-target) resistance mechanisms, detected in approximately 25% of osimertinib-resistant NSCLC. Noteworthy, variability in diagnostic thresholds, which differ between fluorescence in situ hybridization (FISH) and next-generation sequencing (NGS) platforms, complicates its interpretation and clinical applicability. To address MET-driven resistance, several therapeutic strategies have been explored, including MET-TKIs, antibody–drug conjugates (ADCs), and bispecific monoclonal antibodies, and dual EGFR/MET inhibition has emerged as the most promising strategy. In this context, the bispecific EGFR/MET antibody amivantamab has demonstrated encouraging efficacy, regardless of MET alterations. Furthermore, the combination of the ADC telisotuzumab vedotin and osimertinib has been associated with activity in EGFR-mutant, c-MET protein-overexpressing, osimertinib-resistant NSCLC. Of note, several novel agents and combinations are currently under clinical development. The success of these targeted approaches relies on tissue re-biopsy at progression and accurate molecular profiling. Yet, tumor heterogeneity and procedural limitations may challenge the feasibility of re-biopsy, making biomarker-agnostic strategies viable alternatives.

## 1. Introduction

Lung cancer remains the most common cause of cancer-related death worldwide [1]. Among its various subtypes, adenocarcinoma is the most prevalent; it is frequently diagnosed at an advanced stage, with nearly half of the patients receiving a diagnosis when the cancer is already metastatic. Adenocarcinomas of the lung can be categorized as oncogene-addicted (targetable oncogenes drive tumor progression) or non-oncogene-addicted, with the EGFR tyrosine kinase receptor being the second most common targetable mutation and the third most frequently mutated gene overall (TP53 50–60%, KRAS 25–30%), detected in approximately 10–20% of Caucasian patients and up to 50% of Asian patients. The most frequent alterations include exon 19 in-frame deletions (~60%) and L858R single-nucleotide substitution in exon 21 (~30%), which confer sensitivity to known EGFR tyrosine kinase inhibitors (TKIs) [2].

Currently, osimertinib, a third-generation EGFR TKI, is the standard of care for first-line therapy in patients with EGFR-mutant non-small cell lung cancer (NSCLC) that harbors common EGFR mutations. Initially, the AURA3 trial [3] demonstrated the superiority of osimertinib in comparison to platinum-based doublet chemotherapy in T790M-positive advanced NSCLC in patients progressing on first-line EGFR TKI therapy, and subsequently, the FLAURA trial [4,5] demonstrated its superiority over the earlier-generation TKIs in the first-line setting by significantly prolonging both progression-free survival (PFS) and overall survival (OS) while maintaining a tolerable safety profile. Moreover, it is also currently approved for use in the adjuvant setting, prolonging the OS in early and locally advanced disease stages (ADAURA trial [6,7]). Despite the significant responses gained by osimertinib treatment, nearly all patients develop acquired resistance. Indeed, following prolonged treatment, tumor cells compensate by turning to alternative mechanisms in order to circumvent pharmacological pressures, resulting in acquired resistance [8,9,10,11,12,13,14,15,16,17].

The mesenchymal–epithelial transition (*MET*) proto-oncogene was first identified in 1984 through a *TPR-MET* fusion that induced cellular transformation via constitutive kinase activation [18]. That same year, its ligand, hepatocyte growth factor (HGF), was isolated as a mitogen involved in liver regeneration [19]. The *MET* gene is located on the long arm of human chromosome 7 (7q21–31), encodes a heterodimeric, single-pass transmembrane receptor tyrosine kinase composed of extracellular, transmembrane, and intracellular domains, and is activated upon HGF binding, resulting in *MET* homodimerization and the subsequent phosphorylation of specific tyrosine residues of the C-terminal intracellular domain [20,21]. In pathological conditions like inflammation and cancer, HGF is proteolytically activated in the stroma and engages *MET* signaling [22,23], resulting in the activation of several downstream signaling pathways, including the mitogen-activated protein kinase (MAPK)/extracellular signal-regulated kinase (ERK), phosphatidylinositol 3-kinase (PI3K)/protein kinase B (PKB), mammalian target of rapamycin (mTOR), and Janus kinase (JAK)/signal transducer and activator of transcription (STAT) pathways, which promote cell migration, proliferation, and survival [24,25].

The MET receptor is frequently found to be dysregulated in NSCLC patients and can drive cancer progression through various mechanisms. The most common ones are gene amplification, protein overexpression, and exon 14 skipping mutations (*MET*ex14), which are not only seen in de novo in tumors (2–5% for amplification, 2–4% for *MET*ex14) but also (*MET* amplification) in 10–20% of patients with acquired resistance to EGFR or ALK inhibitors [26,27,28]. Concerning acquired resistance to osimertinib, the amplification of the *MET* gene or MET protein overexpression have emerged as two of the most frequent bypass off-target mechanisms, occurring in approximately 25% of cases [29,30].

Therapeutic strategies to overcome *MET*-driven resistance to osimertinib are currently under active investigation. Among these, the dual inhibition of EGFR and MET is achieved through various approaches that involve combinations of small-molecule tyrosine kinase inhibitors (TKIs), MET-directed antibody–drug conjugates (ADCs), and bispecific monoclonal antibodies.

The aim of this review is to contextualize the role and impact of *MET*-driven resistance within the resistance mechanisms to osimertinib, focusing on treatment strategies that are being investigated specifically in this setting.

## 2. Mechanisms of Resistance to Osimertinib (Third-Generation EGFR TKI)

Despite the immense success of osimertinib in first- and second-line treatment, patients with EGFR-mutant NSCLC typically experience disease progression within two years of initiating therapy due to evolving resistance mechanisms that can be broadly categorized into acquired and intrinsic types [30].

Intrinsic resistance refers to altered molecular factors that preexist within tumor cells and are independent of the drug’s target, resulting, in most cases, in immediate resistance and short response durations. Examples of mutations that may play a role in this particular setting are cell cycle proteins, CDK4, CDK6, CDKN2A, and RB1 [31,32,33].

Acquired resistance mechanisms can be classified as either EGFR-dependent (on target) alterations that involve the *EGFR* gene itself or EGFR-independent (off-target) alterations that involve aberrant downstream *EGFR* signaling, the activation of distinct bypass signaling pathways, and histologic transformation [30,34].

Nonetheless, as tumors are highly dynamic and heterogeneous systems, it is not surprising that both mechanisms can be found to co-exist within the same tumor, playing a synergic role in its evolution [10,34]. Whereas patients receiving first- or second-generation TKIs predominantly develop EGFR-dependent mutations (p.T790M missense variant), EGFR-dependent resistance mechanisms are only observed in 10–15% and 20% of patients treated with third-generation TKI osimertinib, either as a first- or second-line treatment, respectively [8,35,36].

The aberrant activation of key downstream EGFR signaling pathways like PI3K/AKT/mTOR and RAS/RAF/MAPK is one way of conferring off-target resistance to osimertinib [30,31,37].

Another resistance mechanism is the histologic transformation of NSCLC into squamous cell carcinoma or small-cell lung cancer, which is correlated with poor prognosis [38,39].

However, bypassing EGFR inhibition remains the most common EGFR-independent acquired resistance mechanism, including HER2 amplification (2–5% of patients receiving osimertinib) [35,40].

Nonetheless, MET receptor signaling is commonly the most frequently altered pathway following EGFR TKI therapy, regardless of the line of treatment received [35,36,41].

## 3. *MET* Gene Amplification vs. Protein Overexpression vs. Exon 14 Skipping

The *MET* oncogene can be dysregulated by several distinct mechanisms, with diverse biological behaviors and diagnostic and therapeutic implications. The most common *MET* alterations observed in NSCLC include *MET* gene amplification, MET protein overexpression, and *MET* exon 14 skipping mutations [42,43].

*MET* amplifications are characterized by an increase in the *MET* gene copy number, resulting in enhanced transcription and subsequent receptor overexpression on cell membranes. They can arise either de novo as primary oncogenic driver mutations or as part of an acquired resistance mechanism.

Ultimately, the ligand-independent activation of MET occurs either via heterodimerization, ERBB3, or homodimerization, resulting in the activation of PI3k/AKT and RAS/MAPK downstream signaling pathways [44,45]. Furthermore, *MET* increases in the gene copy number (GCN) can occur with polyploidy, the duplication of chromosomes, with multiple copies of chromosome 7 (where the *MET* gene resides) present in tumor cells [45,46]. Polyploidy, however, is not a driving gene in biology [47].

MET protein overexpression is associated with either *MET* gene amplification, transcriptional upregulation, or post-transcriptional regulation alterations that favor enhanced protein production [48]. It is detected by immunohistochemical methods and is often graded on a 0–3+ scale, but its clinical relevance as a standalone predictive biomarker remains controversial. Due to differences in antibodies and thresholds, the proportion of MET overexpression in NSCLCs has varied significantly in different studies, ranging from 15 to 70% [49].

*MET* exon 14 skipping mutations are activating alterations that result from the loss of the CBL E3 ligase binding site located at the juxtamembrane domain of the intracellular portion of the receptor, which is normally necessary for appropriate post-transcriptional splicing. Normally, casitas B-lineage lymphoma (CBL), an E3 ubiquitin–protein ligase, is recruited and binds to the phosphorylated tyrosine residue of the juxtamembrane part of the receptor, which subsequently, by ubiquitinating specific residues, bridges MET and endocytic adaptors, ultimately resulting in MET receptor endocytosis and lysosomal degradation. Therefore, skipping exon 14 mutations leads to increased MET receptor stability and prolonged signaling [50]. Unlike *MET* gene amplifications and protein overexpressions, *MET* exon 14 skipping mutations are only found as a primary oncogenic driver in around 3–4% of NSCLC patients, particularly in older patients with sarcomatoid histology [51], and are not reported as resistance mechanisms of EGFR TKIs.

Heterogeneous diagnostic techniques make identifying and interpreting *MET* amplification challenging. Indeed, the definition of *MET* amplification has varied among clinical trials using fluorescence in situ hybridization (FISH) testing (*MET* to chromosome 7 centromere ratio of ≥1.8 to ≥3.0–GCN ≥5 to ≥10) or NGS (tissue: GCN ≥6; liquid biopsy: GCN ≥2.1 to >5) [52].

## 4. EGFR-MET Crosstalk

The EGFR and the MET receptor are two tyrosine kinase receptors that both activate overlapping signaling pathways, including PI3K/AKT, RAS/RAF/MEK/ERK, and STAT3, which converge on common intracellular effectors, all of which mediate cell survival, proliferation, and motility [44,53].

**Figure 1:**
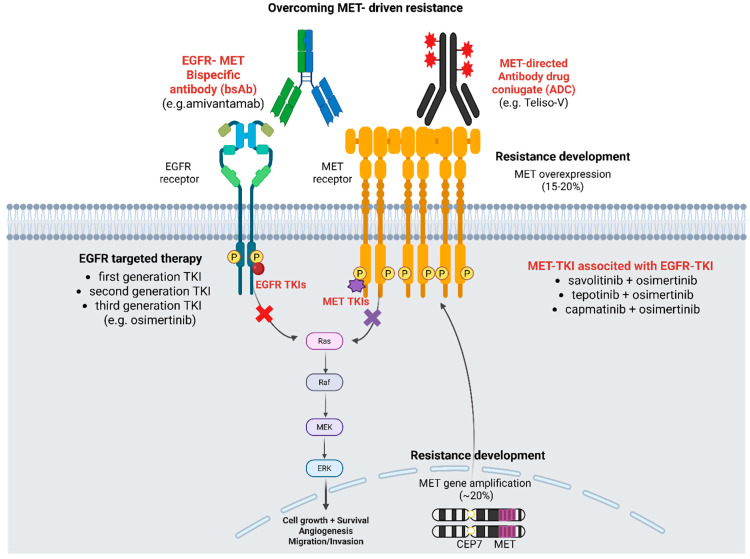
***MET*-induced resistance after EGFR TKI treatment in NSCLC and pharmacological strategies to overcome resistance.** Illustrates the mechanisms of *MET*-driven resistance (protein overexpression and/or gene amplification) post-EGFR TKI therapy. The main strategies to try to overcome this type of resistance are also represented: dual-blockade MET TKI plus EGFR TKI; bispecific anti-EGFR/anti-MET antibodies (e.g., amivantamab), and drug-conjugated antibodies (e.g., Teliso-V). Abbreviations: *MET* = mesenchymal–epithelial transition; RAS = rat sarcoma virus proteins.); Raf = serine/threonine-protein kinase (v-Raf murine sarcoma viral oncogene homolog); P= phosphate; *MEK* = mitogen-activated protein kinase kinase; *ERK* = extracellular signal-regulated kinases; *EGFR* = epidermal growth factor; CEP7: centromere of chromosome 7; TKI(s) = tyrosine kinase inhibitor (s); BsAB = bispecific antibodies; ADC = drug-conjugated antibodies. Created in BioRender. Pamela Trillo Aliaga. (2025). https://app.biorender.com/illustrations/6834acef6b644c3b97443ec2 (accessed on 24 June 2025).

MET receptor signaling is the most frequently altered off-target pathway in the osimertinib resistance setting (Figure 1) [30]. Although the aberrant signaling of this pathway can occur in multiple ways, such as via the paracrine or autocrine overexpression of the receptor’s cognate ligand, HGF, or mutations of the gene itself [41,54], the most frequent and clinically relevant alteration conferring resistance to osimertinib is *MET* gene amplification [35,55]. *MET* amplification was identified in 5–22% of patients progressing on first-generation EGFR TKIs [8,30], while in the subgroup of patients who experienced disease progression to osimertinib, evaluated using liquid biopsy, it was observed in 10% and 10–19% of patients receiving it as a first- or second-line therapy, respectively [35,55,56]. In the AURA study, *MET* amplification was found in nearly 19% of tissue samples from patients resistant to second-line osimertinib. Within this subgroup, MET amplification co-existed with a C797S mutation in 7% of patients [3].

Conversely, c-Met protein overexpression is found in ∼50% of *EGFR*-mutated NSCLC tumors after the progression of EGFR TKIs [57]. However, in the setting of *MET*-driven osimertinib resistance, more robust activity with the combination of MET TKI and EGFR TKI is obtained when MET protein overexpression is primarily the result of *MET* gene amplification [58].

Furthermore, there are suggestions of rare *MET* hot-spot mutations (P97Q, I865F) [59] and other mutations in the MET tyrosine kinase domain (TKD) that may play a role in resistance mechanisms to second-line osimertinib [60]. In contrast, the clear impact of skipping *MET*exon14 mutations in resistance mechanisms remains unknown. Additionally, *MET* gene fusions have been reported as a potential mechanism of osimertinib resistance in some case reports [59,60,61,62,63].

The first experimental evidence of the MET bypass mechanism was elegantly demonstrated in *EGFR*-mutant NSCLC models [64]. The study showed that *MET* amplification results in ERBB3 transphosphorylation, which provides an alternative docking site for PI3K, thereby enabling EGFR-independent pathway activation, even in the presence of gefitinib [64]. Importantly, the inhibition of both EGFR and MET receptors resulted in tumor blockage and even regression, highlighting the therapeutic relevance of dual inhibition.

The initial pre-clinical evidence connecting MET to resistance against third-generation EGFR TKIs came from observations regarding an EGFR-mutant NSCLC cell line, HCC827/ER, with acquired resistance to erlotinib. This cell line exhibited *MET* gene amplification and hyperactivated MET protein and showed cross-resistance to both osimertinib and rociletinib [65]. At the same time, HCC827 cells with acquired resistance to osimertinib (HCC827/AR) also harbored *MET* gene amplification and protein overexpression and were simultaneously resistant to both rociletinib and erlotinib. Additionally, the inhibition of MET with a small-molecule inhibitor or genetically knocking down MET expression restored the ability of osimertinib to inhibit the growth of HCC827/ER and HCC827/AR cells, both in vivo and in vitro, and to inactivate ErbB3 or suppress ErbB3 phosphorylation [65]. Since then, a series of studies have generated similar observations and also demonstrated the significance of dual inhibition in this type of clinical setting, with the potential to overcome this resistance mechanism [66,67,68,69]. These insights have resulted in a series of clinical trials that have evaluated the therapeutic potential of combining EGFR and MET TKIs in EGFR-mutated NSCLC patients who progressed on osimertinib treatment due to acquired *MET* alterations.

## 5. Strategies for Overcoming MET-Driven Resistance to Osimertinib

### 5.1. MET-TKIs

One of the most common off-target resistance mechanisms is *MET* gene amplification, which occurs in approximately 15–20% of patients who progress on osimertinib monotherapy. A series of preclinical studies has demonstrated the significance of the dual inhibition of EGFR and MET in this clinical setting, with the potential to overcome this resistance mechanism [64]. These insights have formulated the rationale for various strategies to manage MET-driven resistance to osimertinib in NSCLC, ranging from biomarker-agnostic to targeted therapies, which will be described in the following sections.

First, MET tyrosine kinase inhibitors (MET TKIs) have been extensively studied to overcome acquired *MET* resistance post-EGFR TKI therapy. Table 1 summarizes the main clinical trials post-osimerinib therapy.

The TATTON trial is an international multi-arm phase 1 study; it was the first to evaluate the dual inhibition of osimertinib and savolitinib in patients with EGFR-mutated advanced NSCLC who experienced disease progression after EGFR-TKI therapy [70,74,75,76]. The trial consisted of four parts (A–D), and based on the safety data obtained from part A, two global expansion cohorts—parts B and D—further examined the combination of osimertinib (80 mg once daily) plus savolitinib (300 mg or 600 mg once daily) in patients with EGFR-mutant, *MET* amplified/overexpressed NSCLC who had progressed on prior EGFR TKI therapy. Part B enrolled 138 patients with three subgroups defined by previous treatment and EGFR-mutation status, who received osimertinib 80 mg once daily plus savolitinib 300 mg or 600 mg once daily. Cohort B1 (69 patients) was previously treated with osimertinib, while B2 and B3 were treated with first- or second-generation EGFR-TKI. Moreover, cohort B2 (51 patients) was T790M-negative, while cohort B3 (18 patients) was T790M-positive. Subsequently, part D was introduced (42 patients), evaluating patients with B2 subgroup characteristics, who received osimertinib 80 mg once daily plus savolitinib 300 mg once daily. *MET* amplification/overexpression was identified either via (FISH; *MET* gene copy number (GCN) ≥ 5 or *MET*–CEP7 ratio ≥ 2) local tissue immunohistochemistry (MET +3 expression in ≥ 50% of tumor cells) or next-generation sequencing (≥20% tumor cells, coverage of ≥200 × sequencing depth and ≥5 copies of *MET* over tumor ploidy) [74]. The final results of the trial were related to the efficacy outcomes: in the subgroups of cohort B, the ORRs were 33% (95% CI, 22–46) for B1, 65% (95% CI, 50–78) for B2, and 67% (95% CI, 41–87) for B3, while for cohort D, it was 62% (95% CI, 46–76). Regarding the median PFS, for the subgroups of cohort B, the median was 5.5 months for B1, 9.1 months for B2, and 11.1 months for B3, while for cohort D, it was 9 months. The median DoR ranged from 9.5 to 11 months across the cohorts, and the longest overall survival (OS) was observed in the B1 subgroup, at 30.3 months (95% CI, 11.8—not calculated). This study confirmed that *MET* amplification/overexpression detected via IHC, FISH, and NGS is a valuable biomarker. Antitumor activity was significant, particularly for patients with *MET* copy numbers ≥10. Furthermore, EGFRm circulating tumor DNA (ct-DNA) clearance on treatment (around cycle 3/4) predicted longer PFS in patients with detectable baseline ctDNA, while nearly half of the patients developed acquired resistance, mainly involving MET, EGFR, and KRAS alterations. The combination treatment of osimertinib plus savolitinib showed a manageable safety profile, with the most common adverse events (AEs) being nausea (36%), peripheral edema (36%), and fatigue (35%); around half of the patients experienced grade ≥ 3 AEs (the most common: neutropenia 7%, pneumonia 7%) [74]. Ultimately, the 300 mg savolitinib dose was better tolerated than the 600 mg one [70]. TATTON ended up serving as a foundational study, highlighting the potential treatment option of dual EGFR and MET inhibition, supporting its evaluation in subsequent clinical trials, such as SAVANNAH, SAFFRON, and INSIGHT 2.

SAVANNAH is an ongoing, single-arm, phase II clinical study investigating the efficacy and safety of osimertinib (80 mg once daily) plus savolitinib (300 mg twice daily) in patients with EGFR-mutant NSCLC and acquired *MET*-mediated resistance to first-line therapy with osimertinib [77]. Initially, the study included patients with *MET* amplification/overexpression (IHC 3+ expression in ≥50% of tumor cells or FISH GCN ≥ 5) in patients on progression on any line of osimertinib (≤3), determined by means of liquid or tissue biopsy and centrally confirmed. The study’s preliminary results showed that patients with high *MET* amplification and/or high overexpression (IHC90+ and/or FISH10+) achieved an ORR of 49%, while patients without high *MET* alterations (<IHC90+ and/or FISH10+) reached only 9% ORR. The median PFS in patients with IHC90+ and/or FISH10+ was 7.1 months, compared to 2.8 months in the subgroup without IHC90+ and/or FISH 10+ [71]. The study’s primary results were recently presented at the 2025 ELCC congress in Paris. A total of 80 patients comprised the primary efficacy population, defined as patients with progression on first-line osimertinib with MET IHC 3 +/≥90% or FISH10+, and achieved an ORR of 56% (95% CI, 45–67%) by investigator assessment (primary endpoint of the study), a median DoR of 7.1 months (95% CI, 5.6–9.6), and a median PFS of 7.4 months (95% CI, 5.5–7.6). Grade ≥ 3 AEs occurred in 57% of patients, with grade 3 or higher treatment-related AEs occurring in 32% and serious AEs occurring in 31% of patients. AEs leading to the discontinuation of savolitinib occurred in 16%, and AEs leading to the discontinuation of osimertinib occurred in 12% of patients. The most common AEs, occurring in at least 20% of patients, were peripheral edema (58%), nausea (45%), diarrhea (33%), and vomiting (21%) [72].

The encouraging results of the SAVANNAH trial led to the initiation of SAFFRON, a global, open-label phase 3 ongoing study designed to compare savolitinb (300 mg twice daily) plus osimertinib (80 mg once daily) to standard platinum-based chemotherapy in patients with locally advanced or metastatic NSCLC progressing after osimertinib (NCT05261399). The study enrolls patients with post-progression tissue biopsy confirmed via central laboratory MET overexpression and/or *MET* amplification (IHC: 90% of tumor cells staining at 3+ intensity or FISH: ≥10 copies of the *MET* gene in tumor cells). The primary endpoint is PFS assessed via blinded independent central review (BICR), while secondary endpoints include OS (in the overall population and in patients with MET overexpression), PFS in patients with MET overexpression, ORR, DoR, pharmacokinetics, and safety. The collected patient samples will be used in exploratory analysis to understand the mechanisms of response and resistance to treatment [78].

INSIGHT is an open-label, phase 1b/2, multicenter, randomized trial study that began in 2013 [58]. The phase 2 study compared the combination of tepotinib plus gefitinib to standard platinum-based chemotherapy in patients who are EGFR-mutant and T790M-negative, with MET overexpression (IHC2+ or IHC3+) and/or amplification (FISH GCN ≥ 5 or *MET*–CEP7 ratio ≥ 2) in advanced or metastatic NSCLC patients who progressed on first- or second-generation EGFR TKIs [58]. The final results were published by Liam et al. in 2023, demonstrating a clear benefit for the combination therapy compared to chemotherapy alone in patients with *MET* amplification [79]. INSIGHT was designed when first- and second-generation EGFR TKIs were part of the frontline treatment; nevertheless, preliminary data were obtained as soon as osimertinib became the new standard of care, leading to the initiation of INSIGHT 2 in 2019, which is a global, two-arm, open-label, phase II trial assessing the efficacy, safety, and tolerability of tepotinib (500 mg once daily) plus osimertinib (80 mg once daily) or tepotinib monotherapy in patients with EGFR-mutated advanced/metastatic NSCLC who progressed on osimertinib and had confirmed *MET* amplification via the FISH method on a tissue biopsy (*MET* GCN of ≥5 or *MET*-CEP7 ratio of ≥2) or via next-generation sequencing on a liquid biopsy (*MET* plasma GCN ≥ 2) [80]. After the protocol amendment, in April 2020, the eligibility was restricted to require progression on first-line osimertinib, the tepotinib monotherapy subgroup was introduced to examine the contribution of osimertinib in this setting, and tissue biopsy FISH (for its higher sensitivity) was also incorporated into central MET testing. The primary analysis of the study was published in August 2024 [73]. Overall, 128 patients were enrolled and initiated on tepotinib plus osimertinib treatment. The primary activity analysis population included 98 patients with *MET* amplification confirmed via central FISH, previous first-line osimertinib, and at least 9 months of follow-up (median 12.7 months). The confirmed ORR was 50.0% (95% CI, 39.7–60.3; 49 of 98 patients), with a median PFS of 5.6 months (95% CI, 4.2–8.1) and median OS of 17.8 months (95% CI, 11.1—not estimable). The most common grade ≥ 3 AEs were peripheral edema (*n* = 6 of 128 patients; 5%), decreased appetite (*n* = 5; 4%), prolonged electrocardiogram QT interval (*n* = 5; 4%), and pneumonitis (*n* = 4; 3%). Serious AEs were reported in 16 patients (13%) [73].

Launched in late 2021, GEOMETRY-E, a randomized, controlled, open-label, multicenter, phase 3 study was designed to evaluate the efficacy and safety of capmatinib plus osimertinib and compare it to the current standard of care treatment of platinum pemetrexed doublet chemotherapy in patients with advanced or metastatic EGFR-mutated, T790M-negative NSCLC with *MET* amplification, who had progressed on either first-, second-, or third-generation EGFR TKI. It consisted of two parts: the first would confirm the safety and tolerability of capmatinib (400 mg twice daily) plus osimertinib (80 mg once daily), while the second would evaluate the efficacy and safety of the combination treatment and compare it to the standard of care chemotherapy regimen [81]. Unfortunately, for business reasons, it was closed preliminarily (NCT04816214). Another phase II trial, LUNG-MAP (NCT05642572), opened, investigating capmatinib with osimertinib with or without ramucirumab in EGFR-mutant, *MET*-amplified stage IV or recurrent NSCLC.

### 5.2. Bispecific Antibody Amivantamab (Anti-EGFR/c-MET)

The bispecific antibody amivantamab simultaneously binds to the EGFR and MET receptors, thus inhibiting ligand binding and promoting cell surface receptor downregulation [82]. Furthermore, amivantamab exerts immune-modulatory effects by triggering antibody-dependent cell-mediated cytotoxicity (ADCC) and Fc-dependent trogocytosis of NSCLC cells [83].

Given amivantamab’s strong affinity for the MET receptor and the role of *MET* amplification as a driver of resistance to third-generation EGFR-TKIs, the combination of amivantamab and EGFR-TKI lazertinib was investigated in EGFR-mutant, osimertinib-resistant, chemotherapy-naïve NSCLC patients in the phase 1 CHRYSALIS trial [84]. In an exploratory analysis, next-generation sequencing (NGS) was used to identify potential predictive biomarkers of response in 45 patients. The combination obtained a higher objective response rate (ORR) in patients with EGFR- and/or *MET*-driven osimertinib resistance (ORR 47%), compared with those with EGFR/MET-independent or unidentified mechanisms (ORR 29%). Notably, the ORR was 61% in cases of *MET* amplification-driven resistance [84].

In cohort D of the CHRYSALIS-2 trial, MET immunohistochemistry (IHC) positivity, defined as MET 3+ staining on at least 25% of tumor cells, was observed in 36% of the available pre-treatment tissue biopsies. MET positivity was associated with higher ORR (61% vs. 14%) and longer progression-free survival (PFS) (12.2 vs. 4.2 months) compared with tumors without MET-IHC positivity [85].

Notably, in the MARIPOSA-2 and MARIPOSA phase 3 trials, amivantamab, combined with platinum-based chemotherapy and lazertinib, respectively, improved clinical outcomes, receiving approval from regulatory agencies regardless of *MET* amplification [86,87,88]. Furthermore, first-line amivantamab was associated with reduced *MET*-driven resistance mechanisms, compared to first-line osimertinib monotherapy, potentially revolutionizing the molecular landscape of resistance to osimertinib [86].

Conversely, the CHRYSALIS trial suggested encouraging the antitumor activity of amivantamab monotherapy in patients with *MET*ex14-driven advanced NSCLC [89,90], with an ORR of 50% in treatment-naïve patients, although these results cannot be translated in the setting of osimertinib resistance [91].

Beyond small-molecule inhibitors and monoclonal antibodies, in recent years, antibody–drug conjugates (ADCs) have gained momentum [92].

The ADC ABBV-399 (Telisotuzumab Vedotin, Teliso-V) includes ABT-700, a humanized IgG1 that targets c-Met, which is conjugated via a cleavable valine–citrulline linker to the cytotoxic microtubule inhibitor monomethylauristatin E (MMAE) [93,94]. In the phase II LUMINOSITY trial, Teliso-V monotherapy was administered to c-MET protein-overexpressing patients with NSCLC. Overall, the highest ORR was observed among patients with c-Met protein-overexpressing, non-squamous EGFR-wild-type NSCLC (37%), and ORR was improved in high-c-Met cases (52%), although PFS and overall survival (OS) were comparable regardless of the level of c-Met expression [95]. In this trial, the ORR for patients with EGFR-mutant NSCLC was 24%, a result that limited the further expansion of this cohort. Nonetheless, a phase I/Ib study (NCT02099058) further investigated Teliso-V both as monotherapy and in combination with other agents, including osimertinib and erlotinib, in patients with advanced solid tumors that were likely to express c-Met. The combination of Teliso-V and erlotinib in patients with c-MET-positive, EGFR-mutant NSCLC who had progressed on prior EGFR-TKI showed antitumor activity, with an ORR of 32.1% in the EGFR-mutant population and 52.6% in high-c-Met tumors, a manageable toxicity profile, and neuropathies being the most common treatment-related adverse event (AE) [96].

In 2023, results were published from arm E of the study, evaluating the combination of Teliso-V and osimertinib in patients with EGFR-mutant, c-MET protein-overexpressing, osimertinib-resistant NSCLC [97]. Among the 38 patients included in the analysis, the c-Met level was high (50–100%, 3+ staining) in 64% and intermediate (25–49%, 3+ staining) in 36% of cases, and in 22% of cases, *MET* amplification was detected via FISH 2+. No dose-limiting toxicities were observed, while 97% of patients experienced AEs; most frequently, these included peripheral sensory neuropathy (50%), nausea (21%), and peripheral edema (21%). Grade ≥ 3 AEs occurred in 32% of patients, including anemia and peripheral motor and sensory neuropathy. AEs leading to Teliso-V discontinuation, interruption, and dose reduction occurred in 24%, 58%, and 37% of patients, respectively. After a median follow-up of 7.4 months, the combination of Teliso-V and osimertinib led to an ORR of ~53% and a median PFS of ~7 months, supporting further evaluation of the combination in the TeliMet NSCLC-03 (NCT06093503) phase 3 clinical trial.

### 5.3. Ongoing Clinical Trials and New Drugs

Table 2 summarizes ongoing clinical trials, along with new drugs and their possible combinations, with the aim of trying to overcome the acquired resistance of *MET* alterations after treatment with EGFR-TKI.

APL-101 (or Vebreltinib or Bozitinib) is a potent and selective small-molecule oral MET inhibitor. APL-101 is being evaluated in a phase I/II study in patients with NSCLC with c-Met dysregulation in advanced solid tumors, specifically in the C2 cohort in patients with EGFR-positive NSCLC who have *MET* amplification as acquired resistance to first-line EGFR inhibitors (Apollomics, NCT03175224). In addition, a combination study with EGFR-TKI is ongoing to assess whether its activity can be enhanced, similar to the TATTON, SAVANNAH, and INSIGHT 2 studies. In a phase Ib/II study, APL-101 in combination with Andatinib (an EGFR-TKI) is ongoing in patients with locally advanced or metastatic NSCLC with MET overexpression or *MET* amplification after failure of treatment with EGFR-TKI (NCT06343064).

Pamvatamig (MCLA-129) is another bispecific IgG1 anti-EGFR/c-MET antibody. In the dose-escalation part of a phase 1/2 study in all solid tumors, the recommended phase 2 dose (RP2D) of MCLA-129 was determined to be 1500 mg every 2 weeks (Q2W) with 28-day cycles (NCT04868877). In the phase II study, MCLA-129 as a monotherapy is used in patients with advanced NSCLC with actionable gene alterations and *MET* amplification (NCT06885840). To enhance the activity of this drug, a phase I study is ongoing that is evaluating MCLA-129 in combination with befotertinib (a third-generation EGFR-TKI) in Cohort B, which includes patients with advanced NSCLC with resistance to third-generation EGFR-TKIs (NCT06015568).

Finally, the combination of amivantamab with other drugs is a strategy to improve acquired resistance to MET. A phase I/II study is evaluating combination therapy with amivantamab and capmatinib (a MET TKI) in cohort 1C with *MET* amplification in previously treated patients with advanced NSCLC (NCT05488314).

## 6. Conclusions

Resistance to third-generation EGFR TKIs, such as osimertinib, remains one of the most significant clinical challenges in the treatment of EGFR-mutated NSCLC. *MET*-dysregulation, specifically through gene amplification or protein overexpression, is identified as the most common off-target resistance mechanism. Recently, advances in molecular diagnostics have enabled the characterization of these types of alterations, specifically at the time of progression, where they can be used as guidelines for treatment selection. This broadens our knowledge of how specific tumors behave, allowing us to develop sophisticated strategies with higher probabilities of success in this type of setting.

Preclinical and clinical evidence support the dual inhibition of MET and EGFR as the most effective strategy currently available. Combinations of bispecific antibodies, such as amivantamab, with third-generation EGFR and MET inhibitors—including osimertinib, lazertinib, capmatinib, tepotinib, and savolitinib—demonstrate encouraging efficacy and tolerability. Data from key studies reinforce the benefit of these approaches in biomarker-selected populations (Figure 1). However, this paradigm relies heavily on accurate molecular profiling, often requiring re-biopsy at progression. The feasibility and clinical utility of re-biopsy remain constrained by tumor heterogeneity, procedural limitations, and access to tissue. In this context, biomarker-agnostic therapies may offer alternative treatment avenues, particularly when re-biopsy is not feasible or biomarker status is indeterminate.

Looking forward, accurately identifying patients who will most likely benefit from such treatments through careful biomarker identification, understanding tumor biology, exploring resistance evolution mechanisms using a primarily system-holistic approach, and combining them with clinical data gained from rationale and thoughtful clinical trials will be critical to improving long-term outcomes. As our understanding and knowledge deepens, integrating emerging therapeutics into personalized treatment algorithms offers us a promising path to overcome *MET*-driven resistance and, most importantly, extend the survival and improve the quality of life of patients.

## Figures and Tables

**Table 1:** Clinical trials using MET TKI plus EGFR TKI in MET-induced resistance post-osimertinib.

Reference	Phase Trial	Setting	MET Testing	Treatment	*n*	ORR (%)	mDoR (mos)	mPFS (mos)	mOS (mos)
Hartmaier 2023 [70] TATTON	Ib-part B1 (dose exp.)	prior 3rd EGFR-TKI	*MET* GCN ≥ 5 or *MET*/CEP7 ratio ≥ 2	osimertinib + savolitinib	69	33 (22–46)	9.5 (4.2–14.7)	5.5 (4.1–7.7)	30.3 (11.8–NC)
	Ib-part B2 (dose exp.)	prior 1st, 2nd EGFR-TKI without T790M	*MET* GCN ≥ 5 or *MET*/CEP7 ratio ≥ 2	osimertinib + savolitinib	51	65 (50–78)	10.7 (6.1–14.8)	9.1 (5.5–12.8)	18.8 (15.1–NC)
	Ib-part B3 (dose exp.)	prior 1st, 2nd EGFR-TKI with T790M	*MET* GCN ≥ 5 or *MET*/CEP7 ratio ≥ 2	osimertinib + savolitinib	18	67 (41–87)	11.0 (2.8–NC)	11.1 (4.1–22.1)	NC (24.4–NC)
	Ib-part D (dose exp.)	prior 1st, 2nd EGFR-TKI withoutT790M	*MET* GCN ≥ 5 or *MET*/CEP7 ratio ≥ 2	osimertinib + savolitinib	42	62 (46–76)	9.7 (4.5–14.3)	9.0 (5.6–12.7)	NC (13–NC)
Ahn 2022 [71] SAVANNAH	II	≥2 L (post-osimertinib)	all IHC50+ or FISH5+	osimertinib + savolitinib	193	32 (26–39)	8.3 (6.9–9.7)	5.3 (4.2–5.8)	na
			with IHC90+ and/or FISH10+	osimertinib + savolitinib	108	49 (39–59)	9.3 (7.6–10.6)	7.1 (5.3–8.0)	na
			without IHC90+ and/or FISH10+	osimertinib + savolitinib	77	9 (4–18)	6.9 (4.1–16.9)	2.8 (2.6–4.3)	na
Ahn 2025 [72] SAVANNAH	II	2 L (post-osimertinib)	IHC 3+/90% and/or FISH10+	osimertinib + savolitinib	80 (inv)	56 (45–67)	7.1 (5.6–9.6)	7.4 (5.5–7.6)	na
Wu 2024 [73] INSIGHT 2	II	2 L (post-osimertinib)	GCN ≥ 5 or *MET*: CEP7 ≥ 2	osimertinib + tepotinib	98	50 (39.7–60.3)	8.5 (6.1–NE)	5.6 (4.2–8.1)	17.8 (11.1–NE)

Abbreviations: MET: mesenchymal–epithelial transition; EGFR: epidermal growth factor receptor; TKI: tyrosine kinase inhibitor; exp.: expansion; Pt. *n*: number of patients; ORR: overall response rate; mDoR: median duration of response; mPFS: median progression-free survival; mOS: median overall survival; mos: months; na: not available; NC: not calculated; NE: not estimable; GCN: gene copy number; CEP7: entromere of chromosome 7 FISH: fluorescence in situ hybridization; IHC: immunohistochemistry.

**Table 2:** Ongoing clinical trials targeting *MET*-driven resistance post-osimertinib treatment in NSCLC.

NCT Number	Drug (s)	Phase	Description	Primary Outcomes	Secondary Outcomes	Status
**Monotherapies**						
**Specific MET TKI**						
NCT03175224 (SPARTA)	Vebreltinib (also known as APL-101, PLB-1001, CBT-101, CBI-3103, bozitinib)	II	Study evaluating APL-101 in subjects with c-*Met* exon 14 skip mutations and c-Met dysregulation in advanced solid tumors (Cohort C2: EGFR-positive NSCLC harboring *MET* amplification as an acquired resistance to first-line EGFR inhibitors)	ORR by BIRC	ORR by inv; DoR, TTP, PFS, OS	Recruiting
**Bispecific Antibodies (Anti-EGFR/c-MET)**						
NCT06885840	Pamvatamig (MCLA-129)	II	Study of MCLA-129 as monotherapy in patients with advanced NSCLC with actionable gene alterations and *MET* amplification (Cohort 1: Patients with *MET* amplification after failure of treatment with EGFR-TKI).	ORR	CBR, DCR, PFS, DoR; TTR; OS, TEAEs, PK, ADA	Not yet recruiting
NCT04868877	Pamvatamig (MCLA-129)	I/II	Dose escalation and expansion study evaluating MCLA-129 in patients with advanced NSCLC and other solid tumors (cohorts also evaluating post-osimertinib resistance).	MTD, RP2D, ORR	BOR, DCR, PFS, OS, safety	Recruiting
**Antibody-Drug Conjugate (ADC)**						
NCT06093503	Telisotuzumab Vedotin (also known as Teliso-V or ABBV-399)	III	Study of Teliso-V (IgG1 monoclonal antibody anti-c-Met bound to the MMAE) combined with osimertinib vs. platinum-based chemotherapy in subjects with c-Met-overexpressing EGFR mutant, locally advanced/metastatic non-squamous NSCLC after a first progression to third-generation EGFR-TKI.	PFS with no CNS metastases at baseline	PFS, ORR, DoR, OS, QoL, safety	Withdrawn (for strategic consideration)
**Combination Therapies**						
**EGFR TKI + MET TKI**						
NCT06343064	Vebreltinib + Andatinib (PLB1004)	Ib/II	Study of Vebreltinib in combination with Andatinib in patients with locally advanced or metastatic NSCLC with MET overexpression or *MET* amplification following EGFR-TKI.	TEAEs, DLT, ORR	PK, safety	Recruiting
NCT04606771	Osimertinib (Tagrisso) + Savolitinib (AZD6094 or HMPL-504)	II	Study of savolitinib in combination with osimertinib vs. savolitinib in combination with placebo in patients with EGFRm+ and *MET*-amplified locally advanced or metastatic NSCLC who have progressed to osimertinib.	ORR by inv	PFS, DoR, OS, ctDNA clearance, PK	Active, not recruiting
NCT05261399 (SAFFRON)	Osimertinib + Savolitinib	III	Study evaluating savolitinib in combination with osimertinib versus platinum-based doublet chemotherapy in patients with EGFR-mutated, MET-overexpressed and/or amplified, locally advanced or metastatic NSCLC who have progressed on treatment with osimertinib.	PFS by BICR	OS, ORR, DCR, DoR, PK, safety	Recruiting
NCT05642572 (Lung-MAP)	Osimertinib + Capmatinib + Ramucirumab *	II	Study of capmatinib plus osimertinib with or without ramucirumab in participants with EGFR-mutant, *MET*-amplified stage IV or recurrent NSCLC.	PFS by inv	DoR, DLT	Recruiting
**Bispecific Antibody (Anti-EGFR/c-MET) + TKI**						
NCT06015568	MCLA-129 (Pamvatamig) + Befotertinib (D-0316) $	I	Study evaluating MCLA-129 combined with befotertinib in patients with advanced NSCLC with EGFR-sensitive mutation (Cohort B patients with 3rd-generation EGFR-TKI resistance).	DLT, MTD, TEAEs	ORR, DoR, PFS, OS, ADA, PK	Not yet recruiting
NCT05488314 (METalmark)	Amivantamab (Rybrevant) + Capmatinib (Tabrecta)	I/II	Study evaluating amivantamab and capmatinib combination therapy in unresectable metastatic NSCLC (Cohort 1C *MET* amplification who have received prior therapy).	Aes, DLT, ORR	DoR, DCR, PFS, OS, TTST; QoL	Active, not recruiting

Legend: * human monoclonal antibody IgG1 anti-VEGFR-2; $ 3rd gen. EGFR-TKI. Abbreviation: NSCLC: non-small-cell lung cancer; TKI: tyrosine kinase inhibitor; MMAE: cytotoxic agent monomethyl auristatin E with microtubule depolymerization activity; ORR: overall response rate; BIRC: blinded independent central review; inv: investigator; DoR: duration of response; DCR: disease control rate; PFS: progression-free survival; OS: overall survival; BOR: best overall response; CBR: clinical benefit rate; TTR: time to response; TTP: time to progression; TEAEs: terms of treatment-emergent adverse event; PK: pharmacokinetics; ADA: anti-drug antibody; MTD: maximum tolerated dose; RP2D: recommended phase 2 dose; QoL: quality of life; TTST: time to subsequent therapy; CNS: central nervous system; ctDNA: circulating tumor deoxyribonucleic acid.
